# P005* PLS3*-associated osteoporosis: a hidden cause of bone fragility in a middle-aged man

**DOI:** 10.1093/rap/rkag113.035

**Published:** 2026-11-03

**Authors:** Katie Feather, Lennart Marahrens, Katie Moss

**Affiliations:** City St George’s, University of London, London, United Kingdom; Department of Rheumatology, St. George’s University Hospital NHS Foundation Trust, London, United Kingdom; Department of Rheumatology, St. George’s University Hospital NHS Foundation Trust, London, United Kingdom; Department of Rheumatology, St. George’s University Hospital NHS Foundation Trust, London, United Kingdom

## Abstract

**Introduction:**

Monogenic causes of osteoporosis are increasingly recognised, with pathogenic variants in *PLS3* emerging as a new cause of X-linked early-onset osteoporosis and recurrent fragility fractures. Diagnosis can be challenging, as affected individuals often lack the characteristic clinical or biochemical features of other inherited skeletal disorders. We report the case of a middle-aged male presenting with multiple fragility fractures, low bone mineral density and positive family history, who was subsequently diagnosed with *PLS3*-related osteoporosis. This case highlights the importance of considering monogenic osteoporosis in young patients with unexplained bone fragility and the value of genetic testing in establishing a definitive diagnosis.

**Case description:**

A 40-year-old man was reviewed for cascade genetic testing by the Clinical Genetics service after an X-linked pathogenic *PLS3* variant, associated with impaired bone mineralisation and skeletal fragility, was identified in his nephew. His nephew had genetic testing after he sustained three low-impact fractures in childhood. There was no family history of coeliac or thyroid disease. The patient reported having gum disease and root canal treatment. Clinical examination revealed no blue sclerae or hearing impairment. Genetic testing subsequently confirmed a hemizygous familial pathogenic *PLS3* variant in the patient. This prompted referral for DXA scan and assessment in the metabolic bone clinic.

An osteoporosis assessment was performed, and he reported a longstanding history of multiple fractures following relatively minor trauma. These included:

• Thumb fracture aged 6,while playing football (managed conservatively);

• Arm fracture aged 11, after falling from a bicycle (managed conservatively);

• Ulna, radius and humeral fractured, aged 23, following an assault (requiring surgical fixation);

• Left proximal humeral fracture, sustained while skiing (managed conservatively);

• Wrist fractures in 2019, after slipping while running (requiring surgical fixation).

He estimated height loss of 2 inches over five years with no history of back pain. Spinal radiographs subsequently demonstrated multiple thoracic vertebral fragility fractures involving T5–T9, while DXA demonstrated severe osteoporosis, with a lumbar spine T-score of -3.8, femoral neck T-score of -2.0, and total hip T-score of -1.8. P1NP, PTH and bone profile were normal, with secondary causes of osteoporosis all negative.

In view of his severe osteoporosis and vertebral fractures, a two-year treatment course of teriparatide was commenced alongside calcium and vitamin D supplementation, to be followed up with a repeat DXA assessment and zoledronic acid.

**Discussion:**

This case highlights the importance of considering both osteoporosis and monogenic causes of skeletal fragility in individuals presenting with recurrent low-trauma fractures, particularly when the fracture burden appears disproportionate to age and conventional risk factors. Despite sustaining multiple fractures throughout childhood and adulthood, resulting in numerous healthcare encounters, no assessment for osteoporosis or secondary causes of bone fragility had been undertaken. A diagnosis was only established following identification of a pathogenic *PLS3* variant in his nephew and subsequent cascade genetic testing.

*PLS3*-related osteoporosis is a rare and likely under-recognised cause of inherited skeletal fragility. Since its first description in 2013, awareness of the condition has increased; however, diagnosis remains challenging. Unlike osteogenesis imperfecta and other inherited bone disorders, individuals with *PLS3* variants often lack characteristic extra-skeletal features such as blue sclerae, dentinogenesis imperfecta and hearing impairment. Family history may also be subtle, particularly given the X-linked inheritance pattern, further contributing to delayed recognition.

This case is particularly relevant as metabolic bone services are likely to encounter increasing numbers of patients with previously unrecognised monogenic causes of osteoporosis as access to genetic testing expands. It also highlights the need to maintain a low threshold for investigating younger adults with recurrent fractures, irrespective of whether a genetic diagnosis is initially suspected.

Management of *PLS3*-related osteoporosis remains challenging due to the limited evidence base and absence of disease-specific treatment guidelines. Given this patient’s severe osteoporosis, vertebral fragility fractures and high future fracture risk, treatment with the osteoanabolic agent teriparatide was initiated to increase bone mineral density and improve skeletal strength. This will be followed by antiresorptive therapy to preserve treatment gains and reduce future fracture risk. Further research is needed to establish optimal long-term treatment strategies and monitoring approaches for patients with *PLS3*-related and other monogenic forms of osteoporosis.

**Key learning points:**

• This case highlights several important lessons regarding the assessment of osteoporosis in younger adults.

• Firstly, not all osteoporosis in young men is secondary in origin. While common causes such as coeliac disease, endocrinopathies, glucocorticoid exposure and inflammatory disease should be excluded, a normal secondary osteoporosis screen should prompt consideration of an underlying genetic cause, particularly in those with recurrent fractures.

• Secondly, vertebral fractures and recurrent low-trauma fractures in younger patients should be regarded as red flags for skeletal fragility. In this case, multiple fractures throughout childhood and adulthood, together with previously unrecognised vertebral compression fractures, ultimately led to the diagnosis. Height loss due to undiagnosed vertebral fractures may be the first clue to a bone disorder and should prompt further investigation irrespective of sex.

• A further learning point is that normal biochemistry does not exclude severe bone disease. Patients with *PLS3*-related osteoporosis may have normal calcium, phosphate, alkaline phosphatase and parathyroid hormone levels, meaning routine laboratory investigations alone may fail to identify the underlying diagnosis.

• This case also reinforces that the absence of classical osteogenesis imperfecta features, such as blue sclerae, hearing impairment or dentinogenesis imperfecta, does not exclude a monogenic cause of bone fragility. Careful evaluation of fracture history and family history remains essential.

• Finally, the case demonstrates the value of genomic testing in unexplained osteoporosis and the potential for earlier diagnosis to shorten the diagnostic journey, facilitate family screening, improve the bone density of carriers and prevent further fractures.

